# Microanatomical and secretory characterization of the salivary gland of the *Rhodnius prolixus* (Hemiptera, Reduviidae, Triatominae), a main vector of Chagas disease

**DOI:** 10.1098/rsob.210028

**Published:** 2021-06-16

**Authors:** Ana Carolina Borella Marfil Anhê, Raquel Soares Maia Godoy, Rafael Nacif-Pimenta, Wagner Faria Barbosa, Marcus Vinicius Lacerda, Wuelton Marcelo Monteiro, Nágila Francinete Costa Secundino, Paulo Filemon Paolucci Pimenta

**Affiliations:** ^1^ Departamento de Engenharia Ambiental, Instituto de Ciências Tecnológicas e Exatas, Universidade Federal do Triângulo Mineiro, Av. Randolfo Borges Júnior, 1400, CEP 38064-200, Uberaba, MG, Brazil; ^2^ Instituto René Rachou, Fundação Oswaldo Cruz, Minas Gerais, Av. Augusto de Lima, 1715, CEP 30190-002, Belo Horizonte, MG, Brazil; ^3^ Departamento de Entomologia, Universidade Federal de Viçosa, Av. PH Holfs, CEP 36570-900, Viçosa, MG, Brazil; ^4^ Fundação de Medicina Tropical Dr. Heitor Vieira Dourado, Av. Pedro Teixeira, 25, Dom Pedro, CEP 69040-000, Manaus, AM, Brazil; ^5^ Programa de Pós-Graduação em Medicina Tropical, Universidade do Estado do Amazonas, Av. Pedro Teixeira, 25, Dom Pedro, CEP 69040-000, Manaus, AM, Brazil

**Keywords:** Rhodniini, vector, Chagas disease, histology, ultrastructure

## Abstract

*Rhodnius prolixus* is the principal vector of *Trypanosoma cruzi*, the aetiological agent of Chagas disease in American countries. This insect is haematophagous during all life cycles and, to antagonize its haemostatic, inflammatory and immune systems, it secretes saliva while feeding on the vertebrate host's blood. Here, we investigated characteristic changes of the salivary glands (SG) that occur during insect development. Two pairs of lobules and ducts comprise the SG of *R. prolixus*. The organ's size increases over time, but the microanatomical structures are preserved during insect development. Both lobules have a single layer epithelium formed by binucleated cells, which surrounds the saliva reservoir. The principal lobule presents higher polysaccharide and total protein contents than the accessory lobe. A network of external muscle layers is responsible for organ contraction and saliva release. Apocrine, merocrine and holocrine secretion types occur in the secretory epithelium. Dopamine, serotonin and tyrosine-hydroxylase are neural-related molecules that regulate SG function both during and after feeding.

## Graphical abstract

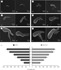

## 1. Introduction

Triatominae (Hemiptera: Reduviidae) are vectors of *Trypanosoma cruzi* (Chagas, 1909), the aetiological agent of Chagas disease. According to estimates, there are 6–7 million individuals infected by this protozoon worldwide, mostly in Latin America, and 90–100 million individuals are exposed to *T. cruzi* infection [1,2]. These insects are vectors of other harmless protozoa, such as *Trypanosoma rangeli* (Tejera, 1920), which have also been described as probably competent vectors of pathogenic bacteria such as *Serratia marcescens* (Bizio, 1923), *Bartonella* (Gieszczykiewicz, 1939) and *Mycobacterium leprae* (Hansen, 1880) [3,4]. Female and male Triatominae individuals are haematophagous during all life cycles, including all five nymphal instars [5]. These insects excrete while feeding on the blood of vertebrate hosts; hence, they can also transmit pathogens to humans through the contact between faeces and the bite site [4–6]. The specific type of saliva, in association with cryptic behaviour, nocturnal habits and mechanical adaptations of mouthparts, allows Triatominae to accomplish many bites, of which few are perceived. These peculiarities provide high blood-feeding efficiency for triatomines [7].

The salivation of Triatominae vectors is activated in the salivary gland (SG) during the entire process of blood-feeding until the final engorgement. Saliva plays a key role in this process, because it is rich in proteolytic proteins, and anti-haemostatic and anticoagulant enzymes. These specific enzymes block coagulation and haemostasis in vertebrate hosts and allow insects to feed [8,9]. The saliva's properties also enable the parasite's penetration and transmission through the bite site [10].

Each insect has one pair of SG located in its thorax. Individuals of the genus *Rhodnius* have two close and independent units forming each SG: (i) a larger and reddish structure called the ‘principal unit’ and (ii) a smaller, translucent, elongated and rounded structure called the ‘accessory unit’ [11–14]. The accessory unit opens at the base of the main excretory duct, which emerges at the medial portion of the principal unit. An accessory duct emerges at the base of the main excretory duct, above the opening of the accessory unit, and connects itself to the digestive tract. The two SG units are formed by a single layer of epithelial cells connected with muscle fibres through a thick tracheole-rich basal lamina [15].

Despite the importance of several triatomines as vectors of human disease and the role of saliva in pathogen transmission, little is known about the biology of the SG, its secretory process, and its morphophysiological changes throughout the life cycle of the insect. The present study adopted conventional histology, histocytochemistry, and morphometry in association with scanning electron microscopy (SEM) and immunofluorescence techniques to better understand the microanatomy and secretory process of SG in the Chagas disease vector *Rhodnius prolixus*. Knowing the secretory dynamics and functioning of the SGs in *R. prolixus*, one of the Chagas disease's primary vectors, can provide additional support for further studies to mitigate transmission and improve to combat this neglected tropical disease.

## 2. Material and methods

### 2.1. Triatomine maintenance and collection

*Rhodnius prolixus* (Honduras strain) samples were provided by *Laboratório de Triatomíneos e Epidemiologia da Doença de Chagas* of *Instituto René Rachou*, FIOCRUZ-MG. Insects were kept under conditions of controlled temperature (26°C) and humidity (55%); first to fifth instar nymphs and adult individuals were collected under two different conditions: (i) Unfed (UF), 7 days after ecdysis; and (ii) blood-fed (BF), 7 days after blood-feeding. The first group of insects was encouraged to feed on blood from anaesthetized mice, but they were stopped before they could bite the mice and were immediately subjected to SG dissection and fixation. The second group of insects was allowed to feed on blood. Engorged insects were interrupted and immediately subjected to SG dissection and fixation.

### 2.2. Salivary gland dissection

*Rhodnius prolixus* individuals were anaesthetized on ice in order to have their SGs dissected. Their thorax was held and their head was pulled off using fine needles under a stereoscope. The exposed SGs were carefully removed, quickly washed in phosphate-buffered saline (PBS, pH 7.4) and fixed using different microscopy techniques.

We analysed 10 glands of each insect stage. All the experiments were realized in triplicates.

### 2.3. Scanning electron microscopy

Dissected SGs were fixed overnight, at room temperature, with 2.5% glutaraldehyde solution with 0.1 M cacodylate buffer at pH 6.8 added. Next, the SGs were post-fixed for 2 h with 2% osmium tetroxide solution with 1.6% potassium ferricyanide added. Samples were dehydrated with a series of increasing acetone concentrations (30–100%) and subjected to critical point drying with CO_2_ [16]. Some dried SGs were fractured to expose their inner side and to allow examination of their secretory surface. Subsequently, SGs were carefully affixed to appropriate SEM stubs and coated with gold particles in a sputtering device. Samples were photographed and analysed using the JEOL 5600 SEM equipment (Tokyo, Japan).

### 2.4. Histology and histochemistry

Dissected SGs were fixed overnight at room temperature with 2.5% glutaraldehyde in 0.1 M cacodylate buffer at pH 6.8. Following this, they were dehydrated using increasing ethanol concentrations (30–100%) for 10 min (each). The samples were then incubated overnight in pure Historesin (Leica Microsystems, Nussloch/Heidelberg, Germany), and, on the following day, they were mixed with hardener (resin/hardener ratio of 15 : 1) to enable the polymerization process at room temperature. Finally, 1 µm-thick sections were cut with the aid of a microtome (Micron HM 340 E). Histological staining was performed with toluidine blue. Histochemistry was performed using periodic acid-Schiff (PAS) [17] and bromophenol blue methods [18]. Stained samples were analysed and photographed using a Zeiss Axiovert 200 M microscope (Konigsallee, Gottingen, Germany).

### 2.5. Morphometric and statistical analyses

The SEM images of the SGs were measured using the SEM analytical software, JEOL JSM 5600 (Tokyo, Japan) to calculate their mean and standard deviations. We analysed 10 glands of each insect stage. All the measures were realized in triplicates. As the principal lobule corresponds to most of the SG, the length of this lobe was used to establish its total size. Nuclei of SGs labelled with DAPI (4′,6-diamidino-2-phenylindole) were counted and measured in five individuals at each stage (nymphs and adults) and condition (UF and BF) using the Image-Pro Plus software (Media Cybernetics). The length of the principal lobe (PL) and the nuclear area of the SG's cells (response variables) were submitted to generalized linear models, considering Gaussian and gamma error distributions, respectively. The explanatory variables were the insect development stage (with six levels) and feeding type (with two levels). Tukey's test was used to discriminate treatments. All analyses were carried out fewer than 5% of significance level using the R software.

### 2.6. Laser scanning confocal and fluorescence microscopy

SGs were fixed with a 4% formaldehyde solution (pH 6) at 4°C for 2 h. After the fixation process was complete, samples were washed for 30 min in RPMI medium (RPMI 1640, Sigma Aldrich) for 2 h and in PBS with 1% bovine serum albumin (PBS/BSA) added. Next, the fixed SG were processed based on the following techniques: (i) SG were incubated for 24 h in rhodamine/phalloidin solution (Molecular Probes; 1 : 1000 in PBS), followed by incubation for 30 min in DAPI solution (Sigma Aldrich), to enable the visualization of their cytoskeleton and nuclei. Control SG were incubated with PBS only. (ii) SG were incubated overnight with the primary antibodies serotonin, tyrosine-hydroxylase and dopamine (1 : 1000 in PBS with 1% BSA) and (iii) phalloidin/rhodamine anti-serotonin to enable immunolabelling of the neurotransmitters and their associated enzymes. (iv) SG were washed in PBS with 1% BSA added and incubated for 4 h with the following secondary antibodies: anti-rabbit-FITC (1 : 100) and tyrosine-hydroxylase (1 : 100) for serotonin; and anti-sheep–FITC for dopamine (1 : 100). All primary and secondary antibodies are derived from Sigma Aldrich. Control groups were incubated with secondary antibodies only. After the fluorescent labelling was complete, all samples were washed three times in PBS (10 min, each) and mounted with Mowiol (Sigma Aldrich) to be analysed using a Zeiss LSM 510 microscope (Konigsallee, Gottingen, Germany). We did two biological replicates and five technical replicates of each insect stage.

## 3. Results

### 3.1. Microanatomy of the *Rhodnius prolixus* salivary gland revealed by scanning electron microscopy

**Figure 1.**
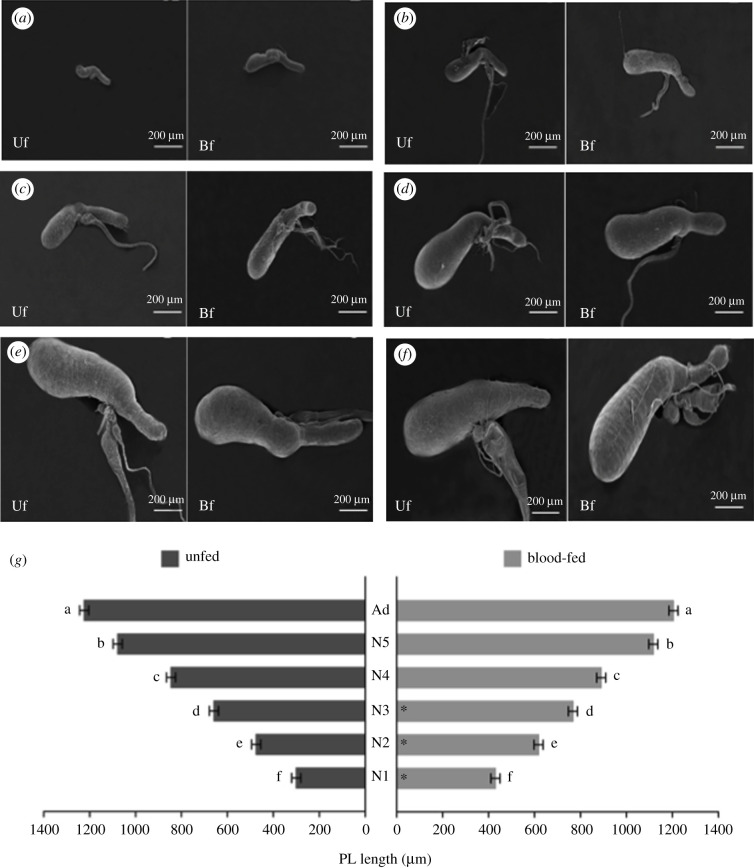
SG of UF and BF *R. prolixus* nymphs and adult. (*a*–*f*) The anatomy of SG remains unchanged throughout the life cycle of *R. prolixus*, but their size increases during insect development. (*a*) First instar, (*b*) second instar, (*c*) third instar, (*d*) fourth instar, (*e*) fifth instar and (*f*) adult individual. (*g*) Lengths of the principal lobule of SGs during insect development. The graph shows the unfolding of the significant interaction (*p* < 0.05) between the explanatory variables used in the model: development stage (with six levels encompassing five juvenile instars plus the adult) and feeding type (with two levels encompassing UF and BF insects). Different letters indicate significant differences among insect development stages within each level of the feeding type using Tukey's test (*p* < 0.05). Asterisks in the bars indicate significant differences between UF and BF insects within each developmental stage of the insect using Tukey's test (*p* < 0.05). When comparing UF and BF insects of the same stage, the length of the PL is higher in BF insects in the N1, N2 and N3 nymphs. By contrast, from N4 to adult, there is no difference in size between UF and BF insects. Regarding insect development, there is a progressive increase in the size from N1 to the adult stage.

The microanatomical structure of the SG in *R. prolixus* is similar at all stages of its life cycle (figure 1*a–f*). SG of the first instar to adult individuals comprise a pair of lobes, which include two lobules named the principal and the accessory lobes. These are linked by their two ducts, the main and accessory ducts. Although the SG's anatomy remains unchanged, the organ grows about four times throughout *R. prolixus* lifetime: first instar = 300 µm and adult = 1205 µm (length of the principal lobule, which corresponds to most of the gland). This increase in the size of the PL occurs progressively from N1 to adult stages. The length of the principal lobule of SG varied between UF and fed insects at N1 to N3 instar nymphs. Fed N1–N3 nymphs presented longer lobules than the UF ones. However, N4–N5 nymphs and adults did not show any differences in the size of SG related to food (figure 1*g*).

**Figure 2.**
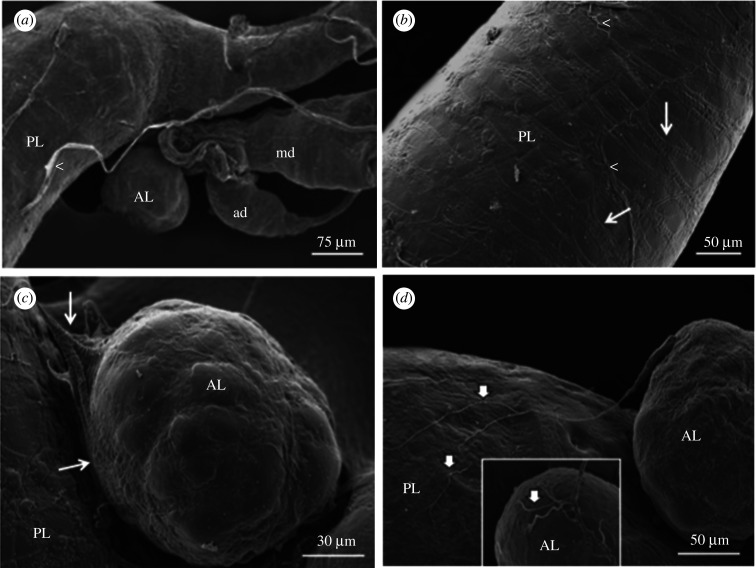
SEM images of the SG in adult *R. prolixus*. (*a*) Medial region of the gland showing principal (PL) and accessory (AL) lobules, main (md) and accessory ducts (ad). (*b*,*c*) Thin arrows show PL (*b*) and AL (*d*) covered by muscle fibres. Ramified tracheoles (arrowheads) can be seen. (*d*) Thick arrows show innervations that go to the accessory lobule and the posterior region of PL (inset).

The principal lobule of the SG is an elongated structure (figure 2*a*), whose medial region houses the accessory lobule and the bases of both main and accessory ducts (figure 2*a*). Several ramified tracheoles are also observed over the lobe surface (figure 2*b*). The accessory lobule (figure 2*c*), which is smaller than the principal lobule (approximately 30 µm in first instar and 150 µm in adult individuals), presents a rounded structure. Transversal anastomosed muscle fibres cover the surface of the principal (figure 2*b*) and accessory lobules (figure 2*c*). Based on figure 2*b*, it is possible to observe that these muscle fibres are flat and have regular striations. It was not possible to discern muscle fibres in the ducts when using the SEM technique (figure 2*a*).

Two nerve cords can be seen in the SG (figure 2*d*). One nerve comes from the duct to the accessory lobule and ramifies into the muscle fibres of the principal lobule. The other comes from the oesophagus to the posterior region of the principal lobule (figure 2*d*, inset).

**Figure 3.**
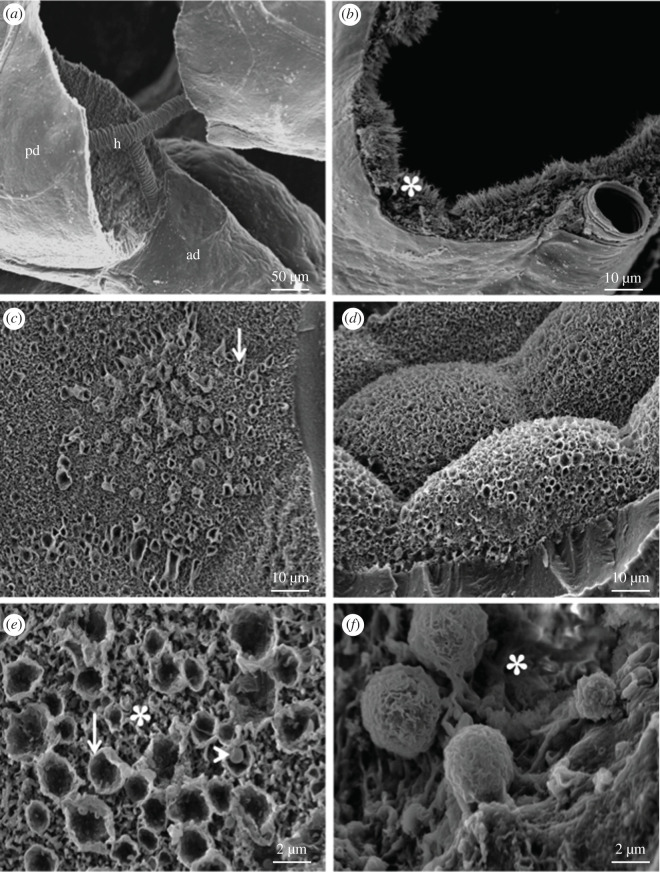
SEM images of the fractured SG of *R. prolixus*. (*a*,*b*) Fractured ducts show the hilum (h) dividing the main (md) and accessory (ad) ducts (*a*) and membrane infoldings (_*_) (*b*). (*c*) The secretory cell's surface is characterized by budding vesicles (arrows). (*d*) Details show the epithelium with a regular aspect, and the hexagonal shapes of the secretory cells. (*e*,*f*) High-magnification views show several open secretory vesicles (arrow) on the cell's surface. Note the presence of small vesicles inside the big ones (arrowhead) and membrane infoldings (*).

Some SGs were fractured to enable analysis of their internal surfaces (figure 3). It was possible seeing a hilum undergoing bifurcation into the principal and accessory ducts when the duct structure was fractured (figure 3*a*). Membrane infoldings with brush aspects are seen inside the ducts (figure 3*b*). When the lobules were opened and their internal surfaces were exposed, it was seen that the secretory epithelium is composed of cells with regular aspects, and most of them present a hexagonal shape (figure 3*c*,*d*).

Membrane infoldings are also seen on the apical surface of the SG's lobules (figure 3*e,f*), but they do not have brush-like aspects as seen in the ducts. Rounded spaces covered by a membrane that resemble open secretory vesicles are detected among the membrane infoldings. Many secretory vesicles are seen above the apical surface of the secretory epithelium (figure 3*e,f*), most of them containing small vesicles inside (figure 3*e*).

### 3.2. Histochemistry and histological characterization of the *Rhodnius prolixus* salivary gland

**Figure 4.**
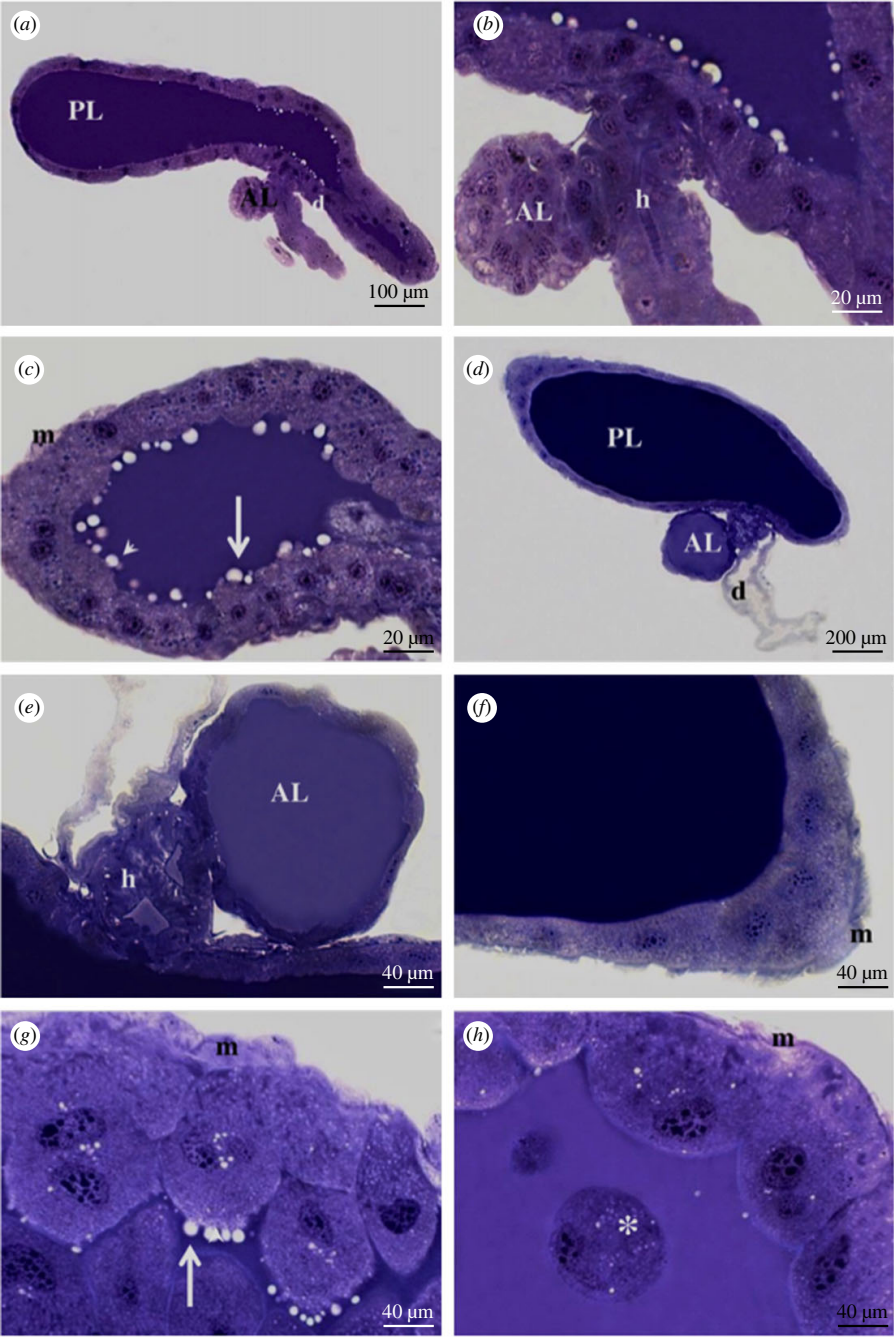
Histological sections of the SG of first instar nymph (*a*–*c*) and adult (*d*–*h*) *R. prolixus* individuals after staining with toluidine blue. (*a*,*d*) Overall view showing the principal (PL) and accessory lobules (AL) and ducts (d). (*b*,*e*) Details showing AL and hilum (h), a common channel between PL and AL that bifurcates into the ducts (d). (*c*,*f*) Details show PL presenting some secretory vesicles (arrows) and muscle fibres (m). (*g*,*h*) Details show cells (arrows) during their release to the PL lumen. Muscle fibres (m) and entire cells detached from the epithelium (*) can also be seen inside the lumen.

Histological analysis of the SG has shown that, throughout the life cycles of *R. prolixus* investigated herein, lobules are formed by a single epithelium covering a large lumen where saliva is stored (figure 4). Toluidine blue staining showed cells in the principal lobule that present rounded-to-oval nuclei, which, in turn, have several rounded regions of condensed chromatin spread over the nucleoplasm (figure 4*c,f*). Unstained vesicles are observed in the lumen of the principal lobule of first instar (figure 4*b,c*) and adult (figure 4*g*) individuals. These vesicles are located close to the internal surface of the glandular epithelium. No vesicles were found in the lumen of the accessory lobule (figure 4*b,e*). In addition, it was observed that the ducts flow into a common channel, namely the hilum (figure 4*b,e*).

Regarding the structure of the epithelial cells of the SG lobules, it was possible to note differences in nucleolar corpuscles during the insect's development. The first life stages were generally characterized by a big central nucleolus surrounded by smaller ones, which, in turn, were close to the border of the nuclear edge (figure 4*a–c*). The latter stage (adult) was mainly characterized by several small nucleolar corpuscles seen in a more central position in the nuclei (figure 4*d,f–h*). Whole epithelial cells were observed in the lumen of adult SGs, but they were not detected in any of the instar nymphs (figure 4*h*).

**Figure 5.**
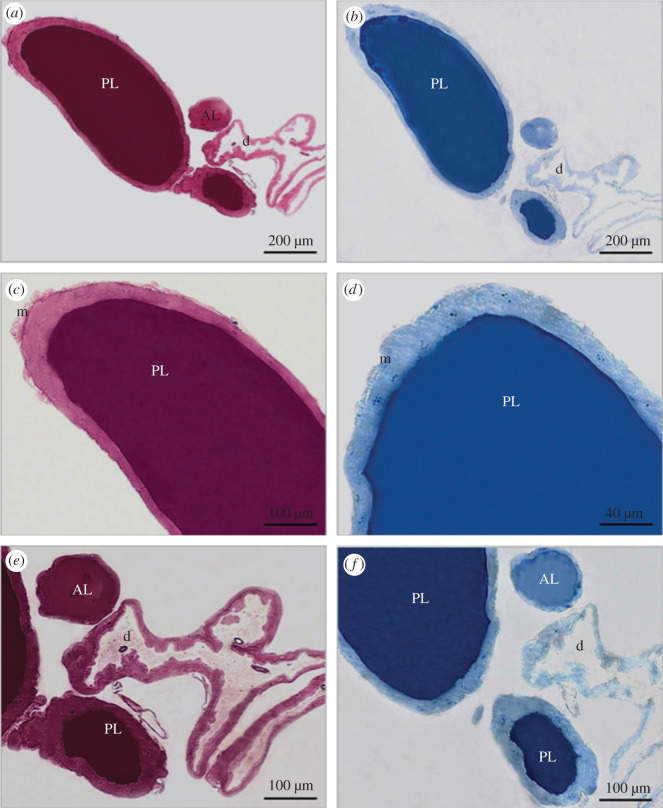
Histochemistry of the SGs of *R. prolixus* individuals after staining with PAS (*a*,*c*,*e*) and bromophenol blue (*b*,*d*,*f*). (*a*,*b*) Overall view of SGs showing stronger staining in the luminal content of the PL than in the AL. (*c*–*f*) Details show PL (*c*,*d*), AL and ducts (*e*,*f*).

When the tissue structures of the two SG lobules in the adult stage of *R. prolixus*, stained by toluidine blue (figure 4*d–f*), bromophenol blue (figure 5*a*,*c,e*) and PAS (figure 5*b*,*d,f*), were compared, the luminal content of the principal lobule presents stronger labelling than the accessory one.

Histological and histochemical changes were not observed between UF and fed insects in any of the life cycle stages of *R. prolixus*.

### 3.3. Fluorescent labelling of muscle fibres and cell nuclei in the whole salivary gland

**Figure 6.**
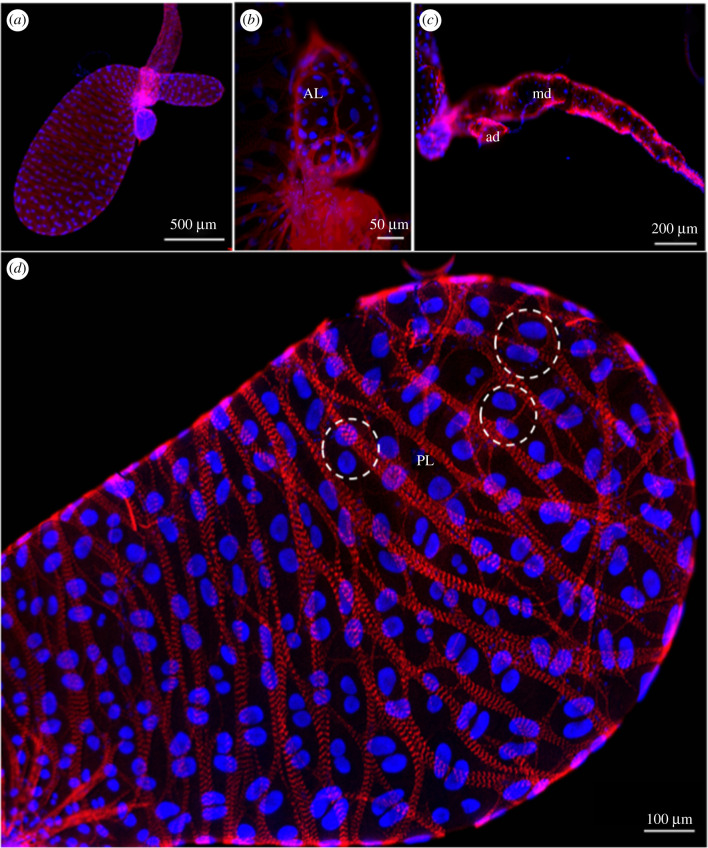
Muscular framework and arrangement of epithelial cell nuclei in the SGs of *R. prolixus*. (*a*) Overall view. (*b*) Accessory lobule (AL). (*c*) Accessory (ad) and main ducts (md). (*d*) Detail of (*a*) shows muscle fibres (red) externally coating the SG and the nuclear arrangement of epithelial cells, internally located close to the muscles. Each epithelial cell of the SG has two morphologically similar nuclei (blue), whose pairs are marked by dotted circles.

Muscle fibres form a framework that covers the whole SG in *R. prolixus* (figure 6*a*), including the principal and accessory lobules (figure 6*a,b*), as well as the ducts (figure 6*c*). Figure 6*d* shows in detail the muscle fibres composed of parallel filaments of actin (red) on the outer cover of the gland and the typical organization of the nuclei of SG epithelial cells, which form pairs of two morphologically similar nuclei (blue).

**Figure 7.**
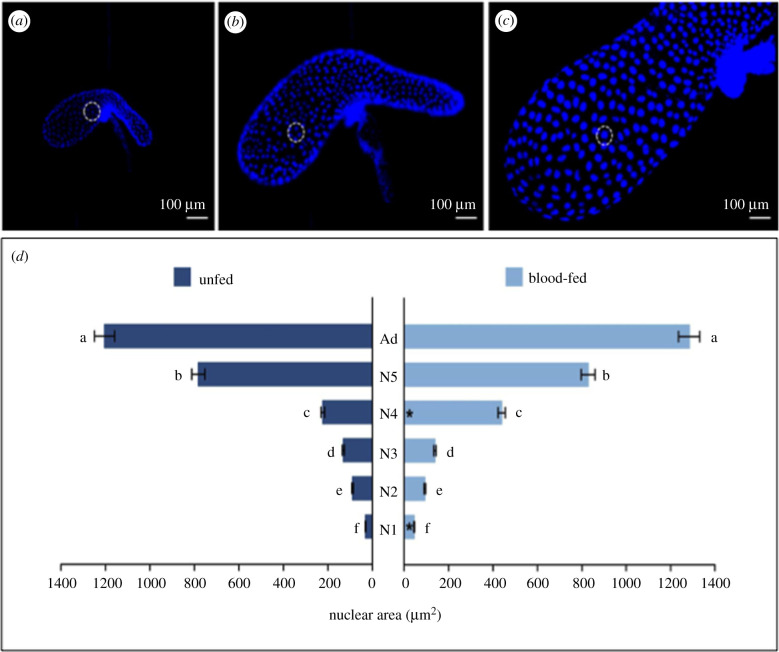
Changes in the nuclear area of cells in the principal lobule of *R. prolixus* SG. Cell nuclei of the principal lobule progressively increased in size from N1 to adult individuals. In (*a*), the dotted circle includes approximately eight nuclei and in (*b*) the number of nuclei is roughly four. In (*c*) the dotted circle of the same size only encompasses one cell nucleus, indicating increased principal lobule nuclear cell size throughout insect development. (*d*) Nuclear area of the cells in the PL of the SG of *R. prolixus*. The image shows the unfolding of the significant interaction (*p* < 0.05) between the explanatory variables used in the model; development stage (with six levels encompassing five juvenile instars plus the adult) and feeding type (with two levels encompassing UF and BF insects). Different letters indicate significant changes among insect development stages within each feeding-type level using Tukey's test (*p* < 0.05). Asterisks indicate a significant difference between UF and BF insects within each insect development stage using Tukey's test (*p* < 0.05). In a comparison between UF and BF insects, the nuclear area of cells in the PL is higher in the BF ones in N1 and N4 instars, but there is no difference related to feeding in the N2, N3 and N5 instars and in adults. Regarding the nuclear area among the distinct stages of insect development, there is a progressive increase in the nuclear area of PL cells from N1 to the adult stage.

SGs labelled by DAPI have also showed a similar number of cell nuclei in the principal lobule throughout insect development (approx. 470). However, the nuclear area of these cells grows approximately 40-fold its original size throughout the insect's life cycle; to about 3 µm^2^ in first instar nymphs until it reaches 1282 µm^2^ in adult individuals (figure 7*a–d*). Additionally, BF individuals of first (N1) and fourth (N4) nymphs presented a nuclear area of PL SG cells greater than those of the UF ones (figure 7*d*).

**Figure 8.**
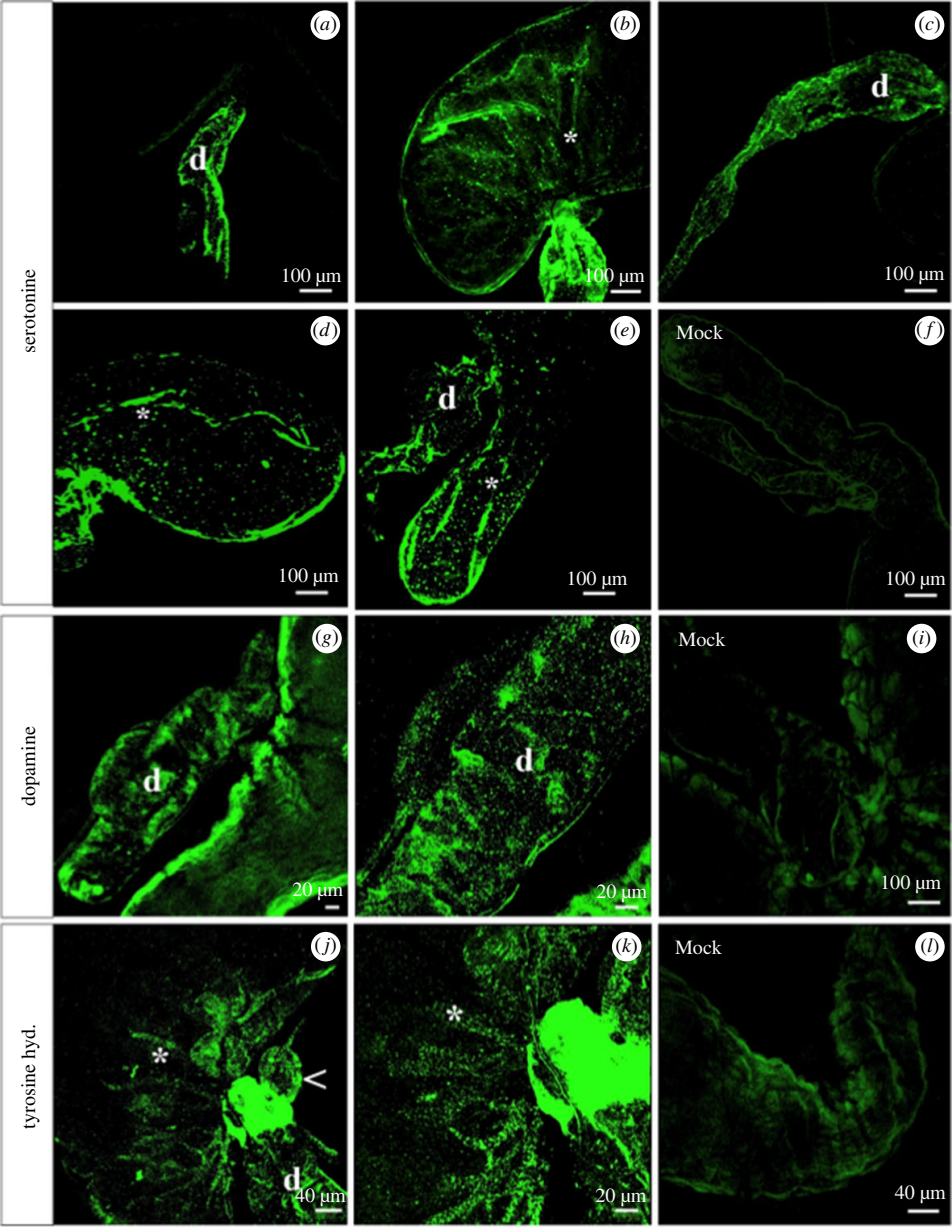
Serotonin (*a–f*), dopamine (*g–i*) and tyrosine-hydroxylase (*j–l*) activity in the SGs of *R. prolixus*. UF insects show serotonergic activity only along the duct of the SG (d). (*b,c*) Insects stimulated with blood meal, 7 days before dissection, show serotonergic activity in the form of linear fibres in the principal lobule (*) and intense activity in the duct (d). (*d–l*) SGs of insects dissected during feeding. (*d,e*) Serotonergic activity is surrounded by a meshwork of serotonergic-branched fibres (*). (*g,h*) Dopaminergic activity is seen in the ducts (d). (*j,k*) Tyrosine-hydroxylase activity in the muscle layers in the middle of the gland (*) and accessory lobule (arrowhead). (*f,i,l*) Control group – SG of insect during feeding incubated only with a secondary antibody. Note a lack of specific labelling.

SG immunofluorescences based on the use of antibodies against neurotransmitters have shown that both UF and fed insects (7 days after blood meal) only presented serotonergic activity in conjunction with the full extension of the ducts (figure 8*a*). By contrast, no tyrosine and dopamine activities were observed in any region of the SG. Insects allowed to feed on blood showed serotonergic activity in linear fibres of the principal lobule (figure 8*b*) and more intense activity in the duct (figure 8*c*).

Insects dissected during blood-feeding showed the entire SG to be surrounded by a meshwork of serotonergic-branched fibres (figure 8*d,e*) with intense activity in the ducts (figure 8*e*). The tyrosine-hydroxylase activity was observed in the entire accessory lobule and in the middle of the principal lobule (figure 8*j,k*). By contrast, the duct presented serotonin (figure 8*e*), dopamine (figure 8*h*) and tyrosine-hydroxylase activity (figure 8*j*). The SGs in the control group showed a total lack of specific activity (figure 8*f*,*i,l*).

**Figure 9.**
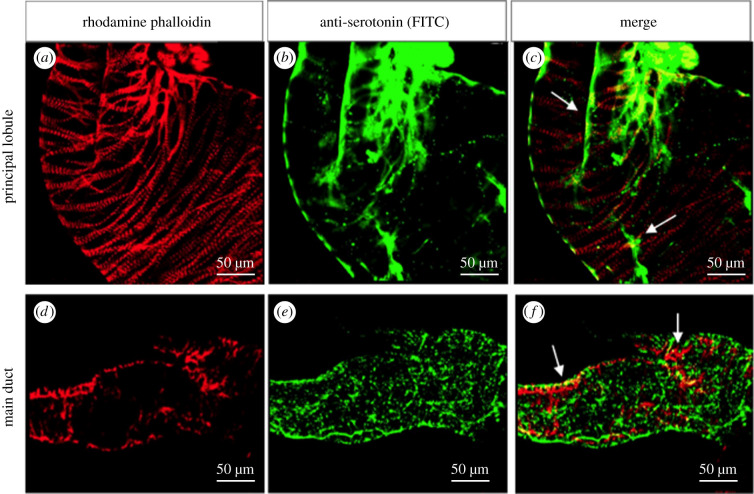
Connections of the serotonin neurotransmitter and the muscle fibres of SG in *R. prolixus*. An association can be seen between the serotonin and muscle fibres (white arrows) in the principal lobule (*a–c*) and the main duct (*d–f*).

The serotonergic fibres were seen along with bundles of muscle fibres in the principal lobule (figure 9*a–c*) and duct (figure 9*d–f*).

## 4. Discussion

SG structure and innervation were substantially similar in all insect life cycles, which may be due to the maintenance of insects' haematophagous dietary habits throughout their growth.

The principal and accessory lobules in the SGs flow into a common channel, namely the hilum. According to Baptist [19], the principal duct of each SG of the pair exits the hilum and goes into the head, where together they form a common short duct (by joining of the two principal ducts), which, in its turn, goes into the salivary pump and opens its distal end. The accessory duct starts at the base of the principal duct and connects itself to the digestive tract in the thoracic region [15]. This structural observation suggests that some salivary secretion can be expelled directly into the oesophagus to make blood ingestion and/or digestion easier. Consequently, the saliva secretory products stored in the lumen of the principal and accessory lobules are mixed in the hilum and divided in order to be secreted in two different places: the salivary pump and the digestive tract.

The SG grows approximately 40-fold throughout *R. prolixus* life cycle. However, the gland maintains the original number of cell nuclei. This indicates that the increase in SG size probably occurs due to cell growth resulting from an increase in cytoplasm volume (several times) over the insect's life cycle, rather than to resulting from cell addition. Accordingly, binucleated cells in the Malpighian tubules of *R. prolixus* grow without adding new cells throughout the cycle [20]. In addition, DNA content in the Malpighian tubule nuclei doubles at each nymph moulting [20]. Hence, it is suggested that DNA replication is achieved through endomitosis; a mechanism adapted to meet the growing insects' excretory needs. Similarly, the same process probably occurs in SG (i.e. increased nuclear content resulting from endomitosis meets the increasing needs for saliva synthesis throughout insect growth). SGs of other arthropods also grow without increasing the total number of cells. The bee species *Bombyx mori* presents two independent SG composed of 330 cells, which grow approximately 1000-fold during larval development [21]. SG mass and protein content in ixodid female tick increase approximately 25-fold during feeding, but the number of cells remains unchanged [22].

BF insects presented longer principal lobules than the UF ones at N1–N3 instar nymphs, although N4–N5 nymphs and adult individuals did not show differences in the organ length of either BF or UF insects. Thus, blood-feeding seems to positively affect the size of SG only in the early stages of insect life, since no such effect was observed on more advanced stages in the life cycle of *R. prolixus*. As an increased organ size may result from the accumulation of salivary secretions, there may have been increased saliva secretion by the epithelium of SG in N1–N3 nymphs during their feeding on blood, a phenomenon that does not occur in N4–N5 nymphs or in adult individuals. However, to confirm this hypothesis, further studies are necessary to understand all the factors responsible for the influence of blood-feeding on the SG's function in the distinct developmental stages of *R. prolixus*, which may also affect on Chagas disease transmission.

A muscular framework was observed covering the SG of *R. prolixus*. These muscles produce gland contraction and saliva release during the feeding process. SG muscular contraction and secretion of salivary components are regulated by neuronal stimuli [23]. The muscle covering has been observed in SGs of other triatomines (*Triatoma infestans*, *Rhodnius domesticus*), as well as in SGs of other insects, such as bed bugs (*Cimex hemipterus*), spittlebugs (*Mahanarva fimbriolata*) and armed spiders (*Phoneutria nigriventer*) [24–26]. Considering that muscle and tracheal cells also cover the salivary duct, it is possible to assume that duct's contractions help the saliva move along the duct, as also suggested by Meirelles *et al*. [15]. In addition, the large number of tracheal cells provides strong organ oxygenation, which is necessary to enable intense metabolism through the gland and muscle activity during saliva secretion [15,27].

Histochemical analyses applied to SGs showed differences between principal and accessory lobules. The secretory material in the lumen of the principal lobule showed stronger staining with PAS and bromophenol blue than in the accessory lobule, which indicates higher neutral polysaccharide and total protein concentrations in the PL. Anhê *et al*. [28] investigated acid phosphatase activity in the SG of *R. prolixus* and *R. neglectus* individuals, and concluded that the accessory lobule presented a less intense reaction than the principal lobule. Acid phosphatases are enzymes found in lysosomes, which are organelles involved in autophagy and cell recycling processes. Therefore, it is possible to assume that the principal lobule of *R. prolixus* SG presents a higher rate of metabolism than the accessory lobule.

Histology and immunofluorescence analyses revealed bulky and polyploid nuclei in the principal lobule. Polyploidy is a common phenomenon in many insect tissues such as in the midgut, epidermis, fat body, Malpighian tubules, trachea and ovary follicles [29]. Beside the polyploidy nuclei, the principal lobule presented binucleated cells at all stages. This increase in nuclear mass indicates an intense rate of metabolism that is capable of accelerating and regulating cellular regeneration after secretion [30]. Ultrastructural analysis indicated that cells are abundant in the endoplasmic reticulum and in mitochondria [15]. These characteristics indicate the high metabolism of cells necessary for saliva production and secretion.

The apical region of epithelial cells in the principal lobule of SG in *R. prolixus* presented membrane infoldings. Cells often increase the membrane area by forming membrane infoldings, which enable a greater total surface area for substance exchange and secretion [11,15]. Some studies have suggested that this mechanism is used for nitric oxide secretion [31] and electrolyte and water transport [32,33], indicating intense secretory activity and molecular exchange in the epithelium of the principal lobule.

Many vesicles were seen sprouting from the epithelial cells in the SG's lobules in a process resembling secretion to the lumen. The ultrastructure showed small vesicles inside the big ones. These vesicles are characteristic of the apocrine secretion type, according to which only part of the cell cytoplasm comprising secretory substances is discarded to the lumen [34,35]. In addition to the vesicles, some entire cells were seen in the lumen. This finding suggested the incidence of the holocrine secretion type, according to which cells accumulate secretory substances, and, in order to release them, it is necessary to have full cell detachment to the lumen [34]. Merocrine secretions, which occur through exocytosis mechanisms, were not identified in the SG of *R. prolixus* individuals investigated in the present study. In regard to other Hemiptera, there were apocrine and merocrine secretions in the SGs of *T. infestans* [11] and *C. hemipterus* [25], and only merocrine secretions in the SG of *R. domesticus* [15]. Therefore, vesicle sprouting that is typical of apocrine secretion appears to be common in the SGs of triatomines. This is the first time holocrine secretion has been seen in the SGs of these insects.

Two points of innervation were identified in the SG of *R. prolixus*. According to Orchard [36], serotonin-like efferent neurons in the sub-oesophageal ganglion pass anteriorly towards the mouthparts, join the salivary nerve and result in refined processes covering the paired SG units. Serotonin is also delivered to SG via the frontal ganglion and the recurrent nerve that projects from the oesophagus. Therefore, nerves connecting to the SG of *R. prolixus* probably derive from the frontal and sub-oesophageal ganglion.

Neurotransmitters, such as serotonin and dopamine, and the neural-related enzyme tyrosine-hydroxylase, presented different intensities and different place labelling in the SG of *R. prolixus*. Serotonin was the most abundant neurotransmitter in the whole of the insect's SG. In general, insects present serotonergic activity before the bite, whereas salivation only occurred after the bite (in the probing and engorgement phases) [37]. This finding suggests that serotonin prepares the gland for the bite by contracting it. These contractions can help mix SG contents and potentially squeeze the saliva out of the principal unit [23]. Salivary ducts showed serotonergic activity in all the analysed *R. prolixus* stages. Serotonin activity has already been identified in salivary ducts of *R. prolixus* [36].

Dopamine and tyrosine-hydroxylase activities were only observed in the SGs of *R. prolixus* that were anaesthetized at the time of feeding on blood followed by immediate gland dissection. Dopamine and tyrosine-hydroxylase activities have been observed in studies conducted with *Periplaneta americana* [38] and *Carausius morosus* [39]. Dopamine has been observed stimulating salivary duct cells in *P. americana*, which suggested that it controls their most likely function (i.e. primary saliva modification). Therefore, dopamine may perform a similar function in the SG of *R. prolixus*.

The SG of *R. prolixus* presented tyrosine-hydroxylase activities in the middle of the PL and the accessory lobule during feeding. Tyrosine-hydroxylase activity is indicative of the presence of catecholamines (dopamine, noradrenaline and adrenaline). Dopamine is considered the principal amine found in insects [40]. However, no dopaminergic activity was observed in the middle of the PL and the accessory lobule, suggesting the presence of other catecholamine types in the tyrosine-hydroxylase-positive neurons of *R. prolixus*.

Finally, it was possible to observe that the SG of *R. prolixus* increased in size, but maintained their initial structure and number of secretory cells throughout insect development—from first instar nymphs to adult individuals. The nervous system acted together with the muscular framework that externally covers the SG's structure in *R. prolixus* and regulates its contraction and saliva release. Furthermore, dopamine, serotonin and tyrosine-hydroxylase are important neural-related molecules that regulate the SG's function in *R. prolixus* during and after feeding. Therefore, knowledge obtained regarding the dynamics and functioning of the SG in one of the main vectors of Chagas disease can support future studies aimed at combating this neglected disease and the spread of human parasites such as the challenging *T. cruzi*. Further studies can help to better understand the likely release of different secretion types by each of the two SG lobules and their salivary compositions, as well as to aid in determining the exact mechanism that regulates the salivary duct function in *R. prolixus*.

## Data accessibility

This article has no additional data.
